# An efficient and flexible framework for inferring global sensitivity of agent-based model parameters

**DOI:** 10.1371/journal.pcbi.1013427

**Published:** 2025-09-08

**Authors:** Daniel R. Bergman, Trachette Jackson, Harsh Vardhan Jain, Kerri-Ann Norton

**Affiliations:** 1 Department of Mathematics, University of Michigan, Ann Arbor, Michigan, United States of America; 2 Department of Oncology, Sidney Kimmel Comprehensive Cancer Center, Johns Hopkins University, Baltimore, Maryland, United States of America; 3 Convergence Institute, Johns Hopkins University, Baltimore, Maryland, United States of America; 4 Department of Mathematics & Statistics, University of Minnesota Duluth, Duluth, Minnesota, United States of America; 5 Program of Computational Sciences, Bard College, Annandale-on-Hudson, New York, United States of America; University of Melbourne, AUSTRALIA

## Abstract

Agent-based models (ABMs) have become essential tools for simulating complex biological, ecological, and social systems where emergent behaviors arise from the interactions among individual agents. Quantifying uncertainty through global sensitivity analysis is crucial for assessing the robustness and reliability of ABM predictions. However, most global sensitivity methods demand substantial computational resources, making them impractical for highly complex models. Here, we introduce SMoRe GloS (Surrogate Modeling for Recapitulating Global Sensitivity), a novel, computationally efficient method for performing global sensitivity analysis of ABMs. By leveraging explicitly formulated surrogate models, SMoRe GloS allows for comprehensive parameter space exploration and uncertainty quantification without sacrificing accuracy. We demonstrate our method’s flexibility by applying it to two biological ABMs: a simple 2D *in vitro* cell proliferation model and a complex 3D vascular tumor growth model. Our results show that SMoRe GloS is compatible with simpler methods like the Morris one-at-a-time method, and more computationally intensive variance-based methods like eFAST. SMoRe GloS accurately recovered global sensitivity indices in each case while achieving substantial speedups, completing analyses in minutes. In contrast, direct implementation of eFAST amounted to several days of CPU time for the complex ABM. Remarkably, our method also estimates sensitivities for ABM parameters representing processes not explicitly included in the surrogate model, further enhancing its utility. By making global sensitivity analysis feasible for computationally expensive models, SMoRe GloS opens up new opportunities for uncertainty quantification in complex systems, allowing for more in depth exploration of model behavior, thereby increasing confidence in model predictions.

## Author summary

Of the variety of computationally complex modeling approaches that exist, agent-based models (ABMs) have emerged as a powerful tool for understanding the interconnected molecular, cellular, and microenvironmental dynamics in health and disease. ABMs are well-suited to capture connectivity and heterogeneity across multiple time and spatial scales in biological and social systems. However, their computational demands escalate and become prohibitive when simulating millions of agents. Fast and accurate methods for global sensitivity analysis and uncertainty quantification are crucial for making ABMs more predictive and reliable. Our approach, Global Sensitivity using Surrogate Models, SMoRe GloS, provides a much-needed flexible, accurate, and efficient platform for exploring parameter uncertainty and sensitivity in complex, multiscale ABMs.

## Introduction

Scientists today are generating abundant data and information as they seek to improve our comprehension of the world around us, revealing the inherent complexity of real-world systems. Agent-based models (ABMs) have emerged as a significant tool for understanding such complex systems, being particularly well-suited to capturing emergent phenomena [1–4]. However, ABMs present significant challenges. The computational cost of running ABMs can become prohibitive when simulating millions of agents [5,6] and the absence of closed-form expressions linking ABM output with input parameters makes it difficult to assess the robustness of results to parameter perturbations [7]. Furthermore, each additional parameter introduces additional uncertainty into the model input space. This uncertainty in model inputs will necessarily propagate to model outputs, raising questions about model accuracy and reliability.

Parameter sensitivity analysis is a widely used technique to quantify uncertainty in model outputs as a function of uncertainty in the inputs, helping us better understand the limitations of the model [8]. This type of analysis identifies which input parameters – and, by extension, the real-world processes they represent – are the most critical determinants of an output of interest [9]. Several methods have been developed for sensitivity analysis in parametric models, including variance-based methods, moment-independent techniques, Monte Carlo methods, and methods using spectral analysis (for recent reviews, see [10,11]).

Simple global sensitivity analysis methods include one-at-a-time methods like the Morris method (MOAT) [12], which is computationally efficient but provides only limited information and is best suited for factor prioritization or preliminary screening of model parameters. For more robust insights, variance-based methods such as the extended Fourier Amplitude Sensitivity Test (eFAST) or Sobol indices are generally preferred. These methods are capable of both factor prioritization and factor fixing but come with a much higher computational cost [9,13,14]. Regression-based methods, like Partial Rank Correlation Coefficient (PRCC), may be employed for factor mapping, which aims to identify important inputs within specific output domains. These methods also have high computational costs [10,15]. Aside from MOAT, the computational expense of simulating complex models remains a major challenge when applying global sensitivity methods to ABMs.

One approach to addressing some of these challenges is to use surrogate models, also known as metamodels or response surfaces. These computationally efficient surrogate models capture the dominant features of complex systems [16], such as ABMs, without the prohibitive computational costs [17–20]. Notably, machine learning (ML) surrogates are gaining popularity [21]. However, they require extensive ABM simulations for training and often offer limited biological interpretability [22].

To mitigate these issues, we have proposed employing *explicitly formulated* surrogate models for approximating ABM behavior and parameterizing computationally complex ABMs with multi-dimensional data [5,6]. This work presents a novel application of this technique to address the critical lack of fast and accurate methods for global sensitivity analysis of large-scale, complex ABMs. Specifically, we develop a new, computationally efficient method, Surrogate Modeling for Recapitulating Global Sensitivity (SMoRe GloS), that uses explicitly formulated surrogate models to infer the global sensitivity of input parameters in ABMs describing complex real-world systems. Our method is agnostic to any specific method for global sensitivity analysis and is easily adapted per user specification. We demonstrate our approach by applying SMoRe GloS to two spatio-temporally resolved ABMs: a 2D *in vitro* cell proliferation model and a 3D vascular tumor growth model. The remainder of this paper outlines how SMoRe GloS computes global sensitivity indices using both efficient and versatile methods, compares these results to direct sensitivity computations, and highlights the computational advantages of our approach.

## Methods

### SMoRe GloS: Surrogate Modeling for Recapitulating Global Sensitivity

Our new method for global analysis of computationally complex models, SMoRe GloS, is implemented in five steps: (1) Generate ABM output; (2) Formulate candidate surrogate models; (3) Select a surrogate model; (4) Infer relationship between surrogate model and ABM parameters; and (5) Use the relationship between surrogate model and ABM parameters to infer global sensitivity of ABM parameters. These are described in further detail below. See SI for details on how we implemented each of these case studies.

For convenience, we introduce the following notation. We will refer to the ABM parameters included in the global sensitivity analysis as ${\vec{p}}_{\text{ABM}}=\langle p_{\text{ABM},1},\ldots ,p_{\text{ABM},m}\rangle$. $\Omega \subseteq {\mathbb{R}}^{m}$, together with a probability distribution *ρ* on Ω, will denote the minimal sample space of ${\vec{p}}_{\text{ABM}}$. Surrogate model parameters will be denoted ${\vec{p}}_{\text{SM}}=\langle p_{\text{SM},1},\ldots ,p_{\text{SM},n}\rangle$. Finally, we will refer to the surrogate model as SM.

**Step 1: Generate ABM output**

Sample ABM parameter values over Ω, making sure to include points along the boundary of Ω, together with some interior points. Aim for good coverage of Ω, bearing in mind the increased computational expense as more parameter values are selected. For this, choose a sampling method appropriate to the model dimensionality and available computational resources–for example, a regular grid, Latin Hypercube Sampling (LHS), or a low-discrepancy sequence such as Sobol sampling–considering each has advantages and disadvantages [23,24]. Next, generate ABM output at each sampled parameter vector, making sure to run multiple simulations in order to get meaningful averaged behavior.

**Step 2: Formulate candidate surrogate models**

Formulate (several) candidate SMs informed by the complex system being studied, the mechanisms encoded within the ABM, the ABM output generated in Step 1, and most importantly, the output metric of interest in which we want to quantify the relative influence of each ABM parameter. More details on formulating explicit SMs are available here: [5,6]. Ideally, arrive at several candidate SMs.

**Step 3: Select a surrogate model**

Select the best candidate from the various SMs formulated in Step 2 as follows. Considering each SM in turn, begin by fitting the SM to ABM output generated at each sampled ABM parameter vector (Step 1). In this process, make sure to collect information on goodness-of-fit of, and uncertainty in, the fitted SM parameters (discussed below). For the given SM, aggregate this information across all ABM output. Repeat this process for every candidate SM.

Goodness-of-fit criteria: Fit the SM to ABM output by maximum likelihood estimation (MLE) [25], weighted least squares optimization [26], or other method of parameter estimation. Record the quality of the fit.

Uncertainty in SM parameters: Quantify the uncertainty in SM parameters by computing confidence bounds when fitting the SM parameters to ABM output generated from each sampled ABM parameter vector. These confidence bounds will be used later, in Step 4. Several methods may be employed for uncertainty quantification (see for instance [16]).

Also quantify how well constrained SM parameters are by noting the span of their confidence bounds. For this, we introduce a metric called the **identifiability index**, which is computed for each SM parameter and reflects whether uncertainty analysis yields upper and/or lower bounds within physically or biologically relevant ranges. Specifically, a parameter receives an identifiability index of 2 if both bounds fall within the relevant range, 1 if only one bound does, and 0 if neither bound is informative. For example, in this work, we use profile likelihoods to assess uncertainty in parameter estimates. A parameter is assigned an index of 0 for a flat profile, 1 for an L-shaped profile, and 2 for a U-shaped profile in the vicinity of its best-fit value (Fig A in S1 Text). For further implementation details, see the SI. In SMoRe GloS, we compute the identifiability index for each SM parameter at every sampled point in ABM parameter space. A high frequency of 2’s indicates that the parameter is consistently well-constrained, whereas a predominance of 0’s suggests unidentifiability, potentially due to an over-parameterized SM.

SM Selection: Select the best SM by considering both the goodness-of-fit and the identifiability index. The goal is to choose an SM that both minimizes residual sum of squares (RSS) scores across ABM output, and has well-constrained SM parameters, as evidenced by a high frequency of 2’s in their identifiability indices. If selecting between SMs with different numbers of free parameters, model selection theory should be applied, for instance, by computing an Information Criterion [27] (see SI for details).

**Step 4: Infer relationship between SM and ABM parameters**

**Fig 1:**
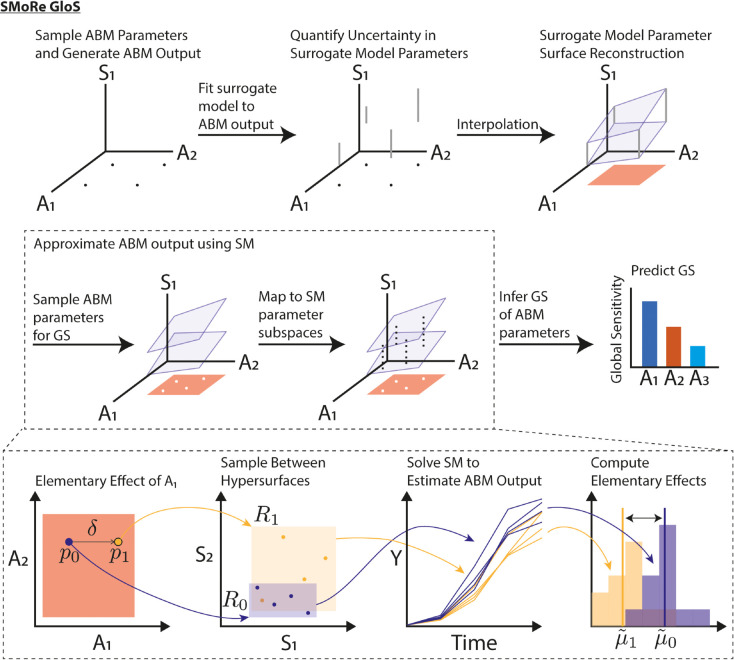
Schematic representation of the SMoRe GloS framework for sensitivity analysis of ABMs. For simplicity, two ABM parameters, *A*_1_ and *A*_2_, and one surrogate model (SM) parameter, *S*_1_, are depicted. The first row shows Steps 1-4 of SMoRe GloS, where *S*_1_ is constrained as a function of *A*_1_ and *A*_2_. The black dots represent sampled ABM parameters, the gray bars indicate uncertainty in *S*_1_ and the blue planes represent the reconstructed parameter surfaces for *S*_1_. The salmon region denotes the interior of the ABM parameter space, defined by the convex hull of the sampled points. The second row illustrates Step 5, where any global sensitivity method can be applied. The white dots represent points in ABM parameter space sampled for computing global sensitivity, and the dashed black lines show the corresponding ranges of *S*_1_. The third row illustrates the implementation of the MOAT method in this framework. Points *p*_0_ and *p*_1_ are examples of white dots from the second row that represent points in ABM parameter space used to compute an elementary effect in *A*_1_. These points correspond to regions *R*_0_ and *R*_1_ in SM parameter space. The time series curves are the trajectories sampled from these regions. The purple and yellow distributions denote the output metric of interest calculated from each trajectory. The elementary effect is approximated by the difference between the means of these distributions.

Quantify the functional relationship between ABM parameters and SM parameters as follows (Fig 1 top row). View each SM parameter as an unknown function – or hypersurface – of the ABM parameters. The (95%) confidence bounds on SM parameters inferred in Step 3 then correspond to discrete points on upper and lower (95%) confidence hypersurfaces ‘above’ the given ABM parameter vector, yielding a range of values for all SM parameters corresponding to each ABM parameter vector. These ranges are usually an interval for each SM parameter. The Cartesian product of these intervals – a hyperrectangle – defines the region of SM parameter space that best fits ABM output at that ABM parameter vector. These Cartesian products quantify the ‘stiff and sloppy’ nature of SM parameters [28], providing information about the directions of SM parameter space that produce small (sloppy) or large (stiff) changes in model behavior. In particular, as the ABM parameter vector is varied, the deformations of these hyperrectangles give rise to variations in ‘stiffness and sloppiness’, which are used to determine ABM parameter sensitivities in Step 5. For more details on how to generate SM parameter hypersurfaces, refer to [5].

**Step 5: Use relationship between surrogate model and ABM parameters to infer global sensitivity of ABM parameters**

Select an output metric of interest, say *f*, on the ABM and a method for computing the global sensitivity of *f* to changes in ABM parameters. *f* is a real-valued function on ABM parameter space, that is, f:Ω→ℝ. The global sensitivity, GS, is then a function of *f* and the probability distribution on ABM parameter space, *ρ*. Denote by $\text{GS}(f(\cdot );\rho )\in {\mathbb{R}}^{m}$ the sensitivity of *f* to each of the *m* varied ABM parameters. The fundamental concept of SMoRe GloS is that an SM is used to estimate *f* in computing GS (Fig 1 middle row). Specifically, the value of *f* at an ABM parameter vector, ${\vec{p}}_{\text{ABM}}$, is approximated by sampling uniformly over the hyperrectangle in SM parameter space in Step 4 above (Fig 1 bottom row). That is,

$$f\left({\vec{p}}_{\text{ABM}}\right)\approx \int_{\Omega_{\text{SM}}({\vec{p}}_{\text{ABM}})}\tilde{f}({\vec{p}}_{\text{SM}})d\mu \left({\vec{p}}_{\text{SM}};{\vec{p}}_{\text{ABM}}\right),$$ (1)

where $\Omega_{\text{SM}}({\vec{p}}_{\text{ABM}})$ is the hyperrectangle in SM parameter space corresponding to ${\vec{p}}_{\text{ABM}}$, $\tilde{f}$ is the functional on SM parameter space to match *f*, and $\mu (\cdot ,{\vec{p}}_{\text{ABM}})$ is the uniform probability distribution on $\Omega_{\text{SM}}({\vec{p}}_{\text{ABM}})$. For notational simplicity, we will use *f* for $\tilde{f}$ and *μ* for $\mu (\cdot ,{\vec{p}}_{\text{ABM}})$ going forward. Putting this together with global sensitivity yields the following:

$$GS(f(\cdot );\rho )\approx GS\left(\int_{\Omega_{\text{SM}}(\cdot )}f({\vec{p}}_{\text{SM}})d\mu \;;\;\rho \right).$$ (2)

Eq 2 is independent of the GSA method used.

### SMoRe GloS case study implementation

We illustrate SMoRe GloS with two ABMs: one describing an *in vitro* cell proliferation assay in 2-dimensions that can be simulated easily and quickly; and one describing vascular tumor growth in 3-dimensions that is computationally complex and more expensive to simulate. Further details, including how SMoRe GloS was implemented for each case, are provided below and in the SI.

**Global Sensitivity Analysis Methods** We demonstrate how SMoRe GloS works using two global sensitivity methods: the one-step-at-a-time Morris method (MOAT), and the variance decomposition-based method, eFAST (extended Fourier amplitude sensitivity test). MOAT perturbs each parameter individually to compute its global sensitivity measure [12,29]. This method has a low computational cost, and its output is in the same units as that of the metric, making the sensitivity indices readily interpretable. Its main limitations are its inability to capture higher-order interactions between model parameters and the fact that it does not yield a definitive boundary separating the important parameters from less influential ones. eFAST estimates the variance of the chosen model output, and the contribution of input parameters as well as their interactions to this variance. The algorithm then separates the output variance into the fraction of the variance that can be explained by variation in each input parameter. The result of this analysis is the main effect and total effect sensitivity indices [13]. eFAST can efficiently handle models with nonlinear responses and complex interactions and is model-independent. However, it is computationally expensive.

**Simple 2D *In Vitro* Cell Proliferation ABM** We first test our new method using the easy-to-simulate ABM presented in [6,30], which describes a 2-dimensional on lattice birth-death-migration model of cell proliferation. This ABM has seven input parameters summarized in Table 1, and is described in further detail in the SI. We infer the global sensitivity of the total cell count at the end of the simulation (*t* = 3 days) with respect to these parameters. To learn the mapping between the ABM and SM parameters, we followed the same steps as previously [6]. In particular, we simulate through Day 3 and record ABM output at set times to match experimental data [31].

**Table 1:** List of ABM and surrogate model (SM) parameters.

2D In Vitro Cell Proliferation Model			
ABM Parameters		SM Parameters (Eqs (3)-(4))	
Name	Meaning	Name	Meaning
*K*_*A*_	Carrying capacity	$\lambda_{C}$	G1/S → G2/M transition rate
$T_{\text{con}}$	Contact inhibition	$\alpha_{C}$	G2/M → G1/S transition rate
*s*	Cell migration rate	*K*_*C*_	Carrying capacity
$\rho_{\text{G1}\to \text{S}}$	G1 → S transition rate		
$\rho_{\text{S}\to \text{G2}}$	S → G2 transition rate		
$\rho_{\text{G2}\to \text{M}}$	G2 → M transition rate		
$\rho_{\text{M}\to \text{G1}}$	M → G1 transition rate		
**3D Vascular Tumor Growth Model**			
**ABM Parameters**		**SM Parameters (Eqs (5)-(4))**	
**Name**	**Meaning**	**Name**	**Meaning**
$p_{\text{div}}$	Progenitor proliferation rate	*λ*	Exponential growth rate
$s_{\text{div}}$	Stem cell proliferation rate	*r*	Logistic growth rate
$r_{\text{mig}}$	Tip cell migration rate	*K*	Logistic carrying capacity
$p_{\text{lim}}$	Progenitor division limit	*α*	vB growth rate
		*β*	vB death rate
		ν	vB exponent

Based on the approach in [6], we chose the following ODE formulation for the SM, with the numbers of cells in G1/S phase (*N*_1*S*_) and G2/M phase (*N*_2*M*_) as model variables.

$$\frac{dN_{1S}}{dt}=-\lambda_{C}N_{1S}+\alpha_{C}\left(2-\frac{N_{1S}+N_{2M}}{K_{C}}\right)N_{2M},$$ (3)

$$\frac{dN_{2M}}{dt}=\lambda_{C}N_{1S}-\alpha_{C}N_{2M}.$$ (4)

For more details on how this SM was derived, see [6]. SM parameters are summarized in Table 1.

**Complex 3D Vascular Tumor Growth ABM** We next test our method on the computationally complex ABM of vascular tumor growth in 3 dimensions presented in [32]. This on-lattice ABM consists of two modules that communicate with each other: a cancer cell module, governing tumor cell proliferation, migration, and death, and a vascular module, governing vascular network growth and remodeling. The ABM has ∼30 input parameters of which four were used in this analysis. These are summarized in Table 1. The ABM is described in further detail in the SI. We infer the global sensitivity of the four ABM parameters with respect to three output metrics: (1) final tumor volume, (2) area under the tumor volume time-course, and (3) time to half-maximum tumor volume.

We chose an ODE formulation for the SM, with the total number of tumor cells (*N*) as the model variable. Three possible formulations were considered for the SM since each of these is a well-established model for tumor growth [33,34]:

$$\text{Exponential Growth}:\;\frac{dN}{dt}=\lambda N,$$ (5)

$$\text{Logistic Growth}:\;\frac{dN}{dt}=rN\left(1-\frac{N}{K}\right),$$ (6)

$$\text{von Bertalanffy Growth}:\;\frac{dN}{dt}=\alpha N^{\theta }-\beta N,\;\theta =1-\frac{1}{\nu },\;\nu >1.$$ (7)

SM parameters appearing in the above equations are summarized in Table 1.

## Results

### Global sensitivity analysis of 2D *in vitro cell proliferation ABM*

**Fig 2:**
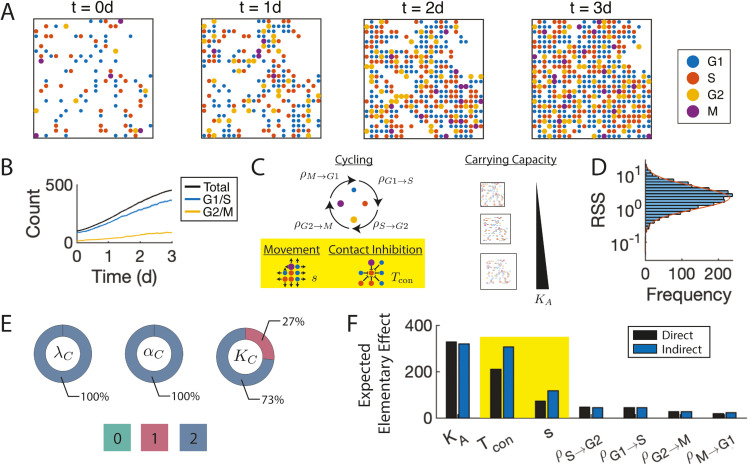
SMoRe GloS recapitulates global sensitivity of cell culture ABM. A) ABM storyboard showing cells by location and cell-cycle phase. B) Time series of the G1/S and G2/M cell-cycle phases. C) ABM parameters included in the sensitivity analysis. The yellow box highlights local spatial parameters that are not explicitly captured by the surrogate model (SM). D) RSS distribution of SM fits to all ABM parameter vectors. Orange line indicates the log-normal distribution that best fits this distribution. E) Identifiability donuts of SM parameters where color indicates the identifiability index, and area the proportion of ABM parameter vectors for which the given SM parameter had that index. F) MOAT sensitivity analysis results using the ABM (Direct, black bars) and SMoRe GloS (Indirect, blue bars), ranked by decreasing sensitivity using the direct method. Spatial parameters not explicitly captured by the SM are highlighted in yellow.

We first implement Steps 1 and 2 of SMoRe GloS, generating output at sampled points in parameter space for the easy-to-run 2D *in vitro* cell proliferation ABM. Fig 2A presents a storyboard showing the spatial and cell cycle phase distributions of cells at various time points during a typical simulation from Day 0 to Day 3. Fig 2B shows time series data of aggregate cell numbers in the G1/S and G2/M phases of the cell cycle from a typical ABM simulation. These data highlight the accumulation of cells in G1/S as the total cell count approaches the carrying capacity and available space is exhausted in the virtual cell culture. ABM parameters, together with the biological processes they regulate, are illustrated in Fig 2C. Parameters that represent spatial processes are highlighted in yellow and include *s*, the rate of cell movement, and *T*_*con*_, the contact inhibition parameter. We note that the surrogate model chosen for this ABM, specified in Eqs (3) and (4), is independent of local spatial considerations and, therefore, does not explicitly incorporate the processes represented by these parameters.

#### Surrogate model accurately matches ABM output with minimal uncertainty in parameter values.

Next, following Step 3 of SMoRe GloS, we calibrate the surrogate model to the ABM output and calculate the residual sum of squares (RSS) to assess the goodness-of-fit. The distribution of RSS values, summarized in Fig 2D, appears log-normal with a low mean (≈1), indicating excellent fit quality. We also apply the profile-likelihood method, as described in Step 3, to quantify uncertainty in the surrogate model parameter estimates. Sample profile likelihood curves for each parameter are shown in Fig B in S1 Text. Across all ABM outputs, $\lambda_{C}$ (G1/S to G2/M transition rate) and $\alpha_{C}$ (G2/M to G1/S transition rate) have well-constrained 95% upper and lower bounds and 100% of their identifiability indices equal to 2 (Fig 2E, first two donuts). *K*_*C*_ (carrying capacity) is well-constrained in the majority of cases. In a smaller number of instances, it is identifiable from only one side, resulting in identifiability indices of 1 (Fig 2E, last donut).

#### SMoRe GloS accurately computes global sensitivity indices of parameters in the 2D *in vitro* cell proliferation ABM, including those not explicitly represented in the surrogate model.

Finally, we implement Steps 4 and 5 of SMoRe GloS to infer the global sensitivity of ABM parameters, using two distinct methods: MOAT and eFAST. In each case, we also directly infer the sensitivities of ABM parameters and use these results to evaluate the efficacy of SMoRe GloS. We present below the results for MOAT. The results for eFAST are similar and can be found in Fig C in S1 Text. We take as our output metric the number of cells in culture at the end of the simulated experiment. Fig 2F compares the global sensitivity of ABM parameters inferred directly (black bars) and indirectly using SMoRe GloS (blue bars) with MOAT. Both approaches produce similar rankings for parameter importance. The direct method indicates higher sensitivity for carrying capacity compared to contact inhibition, although both are deemed highly sensitive by the indirect method. Both methods agree on the insensitivity of transition rates between cell cycle phases and the intermediate sensitivity of cell migration rates. Moreover, relative sensitivities of ABM parameters inferred directly and indirectly are also in excellent agreement (see Fig D in S1 Text).

These results showcase the capability of SMoRe GloS to infer the sensitivity of ABM parameters. Remarkably, this includes parameters representing local spatial processes (highlighted in yellow), such as cell movement and contact inhibition, which are beyond the scope of the surrogate model. It also extends to processes not explicitly included in the surrogate model, such as the transition rates from G1 to S and G2 to M.

### Global sensitivity vascular tumor growth ABM

**Fig 3:**
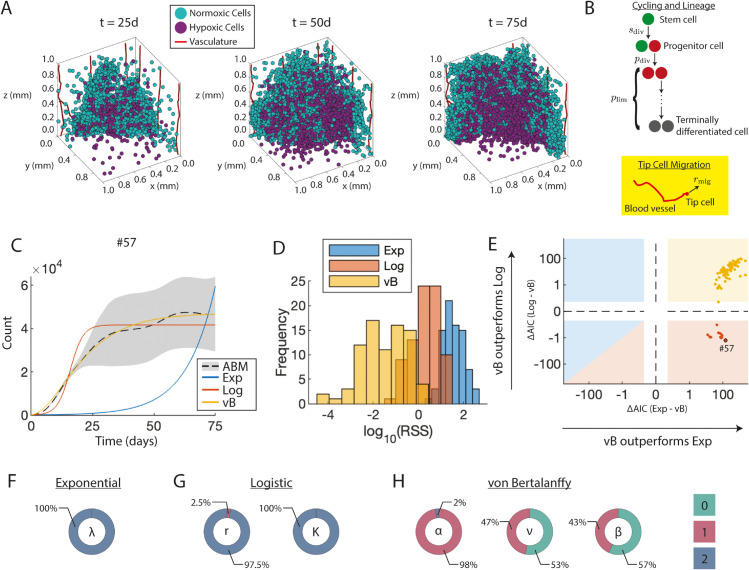
Surrogate model (SM) selection for the 3D vascular tumor growth ABM. A) ABM storyboard showing vascular tumor growth. B) ABM parameters included in the sensitivity analysis. The yellow box highlights local spatial parameters that are not explicitly captured by the surrogate models (SMs). C) Fits of the SMs to ABM output at a representative ABM parameter vector. D) Histograms of log(RSS) values for each SM across all sampled ABM parameter vectors. E) Comparison of Akaike Information Criterion (AIC)-based relative log-likelihoods between the three SMs. Individual ABM parameter vectors are represented as darker colored dots. The x-axis shows the relative log-likelihood of the exponential model, and the y-axis shows the relative log-likelihood of the logistic model, both compared to the von Bertalanffy model. Positive (resp. negative) values indicate that von Bertalanffy is more (resp. less) likely than the alternative SM. The background is color-coded by the SM selected by AIC: yellow indicates preference for von Bertalanffy, red for logistic, and blue for exponential. The ABM parameter vector corresponding to panel C) is highlighted with a black circle. Dashed lines indicate where the log scales change sign. F-H) Identifiability donuts of SM parameters where color indicates the identifiability index, and area the proportion of ABM parameter vectors for which the given SM parameter had that index.

Implementing Step 1 of SMoRe GloS for this case study, we generate output for the computationally complex ABM that models three-dimensional vascular tumor growth. Fig 3A presents a storyboard illustrating the growth of a tumor and its associated vasculature at various time points from a typical simulation. ABM parameters, together with the biological processes they regulate, are depicted in Fig 3B. The rate of tip cell migration parameter $r_{\text{mig}}$ represents a spatial process, and is highlighted in yellow. Following Step 2 of SMoRe GloS, three candidate surrogate models, specified in Eqs (5), (6) and (7)), are chosen for this ABM. Importantly, the parameter $r_{\text{mig}}$ is known to have little effect on tumor growth in this model. We have included it here to assess the ability of SMoRe GloS to perform factor elimination, i.e., identify parameters that have little effect on the model metric.

#### Surrogate model selection for the computationally complex ABM is guided by goodness-of-fit and identifiability indices.

Fig 3C shows average cell number time courses (dashed lines), together with standard deviation (gray shaded area), from ABM simulations generated at a representative set of input parameters. Following Step 3 of SMoRe GloS, this figure also includes fits of the three candidate SMs to the ABM output: exponential growth (blue curve, Eq (5)); logistic growth (red curve, Eq (6)); and von Bertalanffy growth (yellow curve, Eq (7)). Based on visual inspection, the von Bertalanffy model appears to align most closely with the ABM output, whereas the exponential model appears to align least well. This observation is consistent with the quantitative assessment of fit quality based on RSS. The von Bertalanffy model provides the best fit overall, as evidenced by the high frequency of low RSS values and low variance in Fig 3D (yellow histogram), while the exponential model yields the least accurate fits (blue histogram).

The above results are not surprising, given that the exponential model has one free parameter, the logistic model has two, and the von Bertalanffy model has three. To facilitate model selection, the Akaike Information Criterion (AIC) is used to meaningfully compare the fits of the three surrogate models to ABM output, with results summarized in Fig 3E. This figure plots the relative log-likelihood of the von Bertalanffy model compared to the exponential (x-axis) and logistic (y-axis) models. The right half of the figure indicates when von Bertalanffy outperforms the exponential model, while the top half indicates when von Bertalanffy outperforms the logistic model. In particular, the yellow square represents all cases where von Bertalanffy is superior to both the exponential and logistic models (84% of cases). The red square and triangle represent all cases where logistic is superior to both von Bertalanffy and exponential models (16% of cases). In no instance is the exponential model superior to both von Bertalanffy and logistic models (blue square and triangle). The labeled dot corresponds to the ABM parameters whose trajectories are shown in panel C.

Continuing to implement Step 3 of SMoRe GloS, we employ the profile-likelihood method to quantify uncertainty in the parameter values of all three surrogate models. See Fig E in S1 Text for representative profile likelihood curves. Figs 3F-3H show the corresponding identifiability index donut charts for these surrogate model parameters, aggregated over all ABM output. As can be seen, parameters in the exponential model (Fig 3F) and the logistic model (Figs 3G) have identifiability indices of 2 in almost all cases, suggesting these parameters are well constrained by the ABM output. In contrast, the identifiability indices for the von Bertalanffy model parameters *β* and ν are almost evenly distributed between 0’s and 1’s, and almost exclusively 1’s for *α*. This indicates that the von Bertalanffy model parameters are poorly constrained by the ABM output. Thus, even though the von Bertalanffy model provides the best quality of fit, as evidenced by low RSS values, the uncertainty in its parameter values is greatest. On the other hand, while the exponential model has the most tightly constrained parameter estimates, it provides the poorest quality of fit.

Considering these results, we expect the logistic model to perform best in the final step of SMoRe GloS due to its consistently good fits to ABM output and low uncertainty in parameter values. The exponential and von Bertalanffy only meet one of these criteria and are, therefore, not expected to yield optimal results.

#### SMoRe GloS accurately computes the global sensitivity indices of ABM parameters, with one surrogate model emerging as the best choice.

We now proceed to implement Steps 4 and 5 of SMoRe GloS to infer the global sensitivity of ABM parameters, employing two distinct methods: MOAT and eFAST. In each case, we also directly infer the sensitivities of ABM parameters and use these results to evaluate the efficacy of SMoRe GloS. We present below the results for MOAT. The results for eFAST are similar and can be found in Fig F in S1 Text.

**Fig 4:**
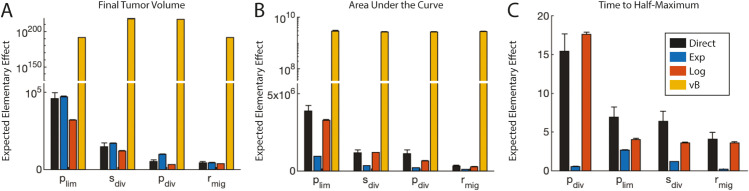
SMoRe GloS recapitulates global sensitivity of multiple output ABM metrics using the logistic surrogate model (SM). Each panel shows the resulting sensitivity values for different output metrics. The colors of the bars correspond to the SM, as shown in the legend in panel (C). A) Using final tumor size as the output metric. B) Using the area under the curve as the output metric. D) Using time to half the maximum tumor volume as the output metric. Note the break in the y-axis scale in A and B.

For the global sensitivity analysis, we use three distinct output metrics to highlight the importance of surrogate model selection in Step 3 of SMoRe GloS: (1) final tumor size, (2) area under the tumor volume time-course curve, and (3) time to half-maximum tumor volume. We selected these metrics for their ability to capture different aspects of the data simulated by the ABM, with the primary aim of emphasizing the importance of surrogate model selection rather than their biological relevance. Fig 4 compares the MOAT-based global sensitivity of ABM parameters across the three output metrics, showing both direct inference (black bars) and SMoRe GloS-based estimates with the three surrogate models: exponential (blue), logistic (red), and von Bertalanffy (yellow). To assess robustness of our method, we computed MOAT-based global sensitivities for each output metric using the exponential and logistic surrogate models at three different ABM sample sizes: 15, 25, and 1000. As shown in Fig G in S1 Text, the inferred sensitivities remained consistent across these sample sizes.

*Selecting a surrogate model solely based on goodness-of-fit to ABM output is insufficient for capturing global sensitivity:* For all three global sensitivity metrics, the von Bertalanffy model – despite its superior fit to the ABM output – fails to adequately capture the sensitivity of the ABM parameters (Figs 4A and 4B, yellow bars). Notably, the results for time-to-half-maximum tumor volume were so poor that they were omitted from the graph (Fig 4C). This poor performance of the von Bertalanffy model is likely attributable to the lack of parameter identifiability, as evidenced by the identifiability indices in Fig 3H. This lack of constraint led to the inclusion of several parameter combinations that produced biologically implausible or unstable outputs. This highlights the limitations of selecting a surrogate model based solely on goodness-of-fit to ABM output without accounting for the risk of over-parameterization. Such an approach can severely compromise the method’s effectiveness.

*Selecting a surrogate model solely based on minimizing uncertainty in its parameters is insufficient for capturing global sensitivity:* The exponential and logistic models effectively predict the global sensitivities of ABM parameters with respect to final tumor size, as shown in Fig 4A (blue and red bars, respectively). The exponential model marginally outperforms the logistic model in capturing the sensitivity of the most significant parameter, while the logistic model excels in predicting the relative sensitivities of ABM parameters (Fig H in S1 Text).

Notably, the exponential model, which has the best identifiability indices, exhibits declining accuracy in calculating global sensitivity as the output metric becomes more reliant on the dynamic aspects of tumor growth. While it can accurately predict the order of importance of ABM parameters for the area under the tumor volume time-course curve (Fig 4B, blue bars), it fails to capture the true sensitivities of these parameters, and completely fails when assessing the time to half maximum tumor volume (Fig 4C, blue bars). This is further evidenced by observing the predicted relative importance of ABM parameters (Fig H in S1 Text).

*Capturing global sensitivity accurately requires balancing good fits to ABM output with minimizing uncertainty in surrogate model parameters:* The logistic model consistently reproduces the sensitivities of ABM parameters across all evaluated metrics (Figs 4A-4C red bars). These findings highlight the critical need to balance maximized goodness-of-fit with minimizing surrogate model parameter uncertainty when performing model selection in Step 3 of SMoRe GloS.

### Computational efficiency of SMoRe GloS for computing global sensitivity

**Fig 5:**
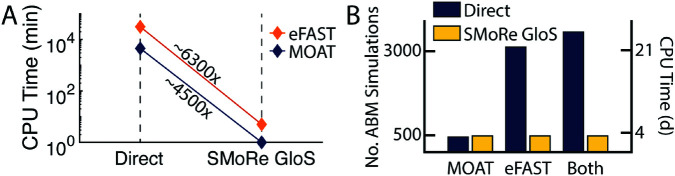
Comparison of ABM simulations and CPU time for computing global sensitivities using MOAT and eFAST in 3D vascular tumor growth ABM. A) Chart showing SMoRe GloS speedup (expressed as times faster) compared to direct implementation of global sensitivity analysis methods. The speedups exclude the setup time for the surrogate model. B) Number of ABM simulations and CPU time required to implement MOAT, eFAST, or both, either directly (blue bars) or with SMoRe GloS (yellow bars), including the setup time for the surrogate model. CPU time is based on assuming 1 ABM simulation takes 10 minutes.

The primary advantage of SMoRe GloS over directly computing global sensitivity with a complex model lies in its significant computational efficiency. To perform the ABM simulations, we used the University of Michigan’s Great Lakes high-performance computing (HPC) cluster, with each simulation run on a single compute node. Due to the shared nature of the cluster, node specifications may vary. The surrogate model was run on a MacBook Pro (13-inch, M1, 2020) equipped with an Apple M1 chip featuring an 8-core CPU and 8 GB of unified memory. Implementing MOAT directly on the 3D vascular tumor growth ABM required 450 ABM simulations, resulting in a total wall time of approximately 75 hours when run serially. In contrast, SMoRe GloS required 7,500 surrogate model simulations, which took less than one minute to run (Fig 5A, blue line). For the more computationally intensive eFAST method, even more ABM simulations were required, further highlighting the efficiency gains of SMoRe GloS. Using eFAST on the same ABM required 3,120 ABM simulations, which, if run serially, results in a wall time of ∼22 days. In contrast, SMoRe GloS once again demonstrated its computational superiority by completing the eFAST analysis in under 5 minutes on a single desktop machine (Fig 5A, orange line).

SMoRe GloS does require an initial investment of computational resources for generating ABM output at sampled points in the ABM parameter space and profiling the surrogate model against this output. For the vascular tumor growth model, these steps required 486 ABM simulations. While this number is comparable to the simulations required for directly computing MOAT sensitivities, it is significantly lower than what would be required for directly implementing eFAST. With just these 486 simulations, SMoRe GloS successfully recapitulated *both* MOAT and eFAST global sensitivity results (see Fig 5B). Furthermore, once SMoRe GloS has been set up, additional global sensitivity analyses using different methods can be performed without incurring further setup costs.

## Discussion

In this paper, we introduce a novel method for inferring the global sensitivity of parameters in agent-based models (ABMs): Surrogate Modeling for Recapitulating Global Sensitivity (SMoRe GloS). This first-of-its-kind approach leverages explicitly formulated surrogate models to approximate ABM outputs, enabling a comprehensive exploration of parameter space that would otherwise be computationally prohibitive. Our findings demonstrate the potential of SMoRe GloS to significantly enhance the efficiency of global sensitivity analysis for ABMs, without compromising accuracy when applied judiciously.

One of the key strengths of SMoRe GloS is its combination of flexibility and adaptability. We demonstrated that our method performs consistently well with both eFAST and MOAT. By being robust to global sensitivity analysis techniques, SMoRe GloS offers greater compatibility across various sensitivity methods, with differing objectives like factor elimination, factor fixing, factor mapping and factor prioritization. This adaptability allows users to tailor the approach to their specific needs and preferences, which is particularly valuable given the wide range of applications for ABMs. Our successful application of SMoRe GloS to both, a two-dimensional cell proliferation assay, and a more complex three-dimensional vascular tumor growth model, highlights its broad utility.

A key design choice in SMoRe GloS is the use of a mechanistic surrogate model rather than a machine learning (ML) model. ML-based surrogates require large numbers of ABM simulations for training, often making them prohibitively expensive. In contrast, mechanistic models generalize more robustly beyond the training set and offer greater interpretability, making them valuable tools for further analysis. For all but the simplest ABMs, deriving a mean-field approximation is infeasible or even impossible. As we show here, such approximations are not required for effective global sensitivity analysis using our approach.

SMoRe GloS offers significant computational efficiency over traditional global sensitivity analysis methods. While direct implementations of these methods often require a large number of ABM simulations and substantial CPU time, we demonstrated how our approach can dramatically reduce both the number of simulations and the computation time. Even after accounting for the initial cost of setting up the surrogate model, SMoRe GloS provides substantial advantages in terms of speed and flexibility. This is especially beneficial for more complex tasks, such as factor mapping and prioritization, which typically have high computational costs.

Additionally, while our implementation used on-grid parameter sampling, which scales exponentially with the dimensionality of the parameter space, further optimizations–such as using Latin Hypercube Sampling (LHS) or Sobol sequences, which scale linearly–could further reduce the computational cost of setting up the surrogate model. This reduction is crucial, as many complex models require significant time per simulation, making direct global sensitivity analysis computationally prohibitive. SMoRe GloS, however, makes such analyses feasible.

Another notable feature of SMoRe GloS is its empirically observed ability to produce global sensitivity indices for ABM parameters that are not explicitly included in the surrogate model formulation. This feature suggests potential utility for complex models where certain biological or real-world processes are difficult to capture with computationally less expensive surrogate models. The implications are significant: in our case studies, we found that SMoRe GloS can accurately compute the sensitivity of spatial parameters that appear in an ABM, even when they are absent from a spatially-independent surrogate model.

One caveat of our approach is that the effectiveness of SMoRe GloS in accurately recovering the correct sensitivity indices of ABM parameters hinges on the choice of surrogate model. To address this, we advocate for a balanced approach to surrogate model selection, guided by both goodness-of-fit to ABM output and the identifiability properties of surrogate model parameters. Specifically, the focus during surrogate model selection should be on ensuring it faithfully reproduces the ABM output with minimal uncertainty. The particular output metrics of interest, for which we wish to determine the sensitivities of ABM parameters, should be considered after selecting a robust surrogate model. Since a well-constrained surrogate model will be broadly applicable, it can effectively assess a variety of output metrics, making our approach particularly valuable given the unpredictable nature of exploratory modeling.

There are several promising avenues for further developing and extending SMoRe GloS. One approach under consideration is to rank ABM parameters based on their influence on surrogate model parameters, which could then be integrated with a sensitivity analysis of the surrogate model to produce a global sensitivity ranking for the ABM parameters. This method may eliminate the need to reconstruct surrogate model parameter hypersurfaces, improving efficiency. Additionally, obtaining a well-constrained surrogate model that accurately reproduces ABM outputs is essential. To enhance this, we are exploring machine learning and equation learning algorithms. These approaches could lead to more robust and accurate surrogate models, ultimately broadening the applicability and efficiency of SMoRe GloS in various complex biological and real-world systems.

## Supplementary Text, Table, and Figure Captions

S1 Text Supplementary information
