# Correction: An efficient and flexible framework for inferring global sensitivity of agent-based model parameters

**DOI:** 10.1371/journal.pcbi.1013613

**Published:** 2025-10-27

**Authors:** Daniel R. Bergman, Trachette Jackson, Harsh Vardhan Jain, Kerri-Ann Norton

An additional affiliation is missing for the third author. Harsh Vardhan Jain is also affiliated with the Department of Mathematical Sciences, Carnegie Mellon University, Pittsburgh, Pennsylvania, United States of America.
