# Supplementary material for: An efficient and flexible framework for inferring global sensitivity of agent-based model parameters
Source: PLoS Comput Biol. 2025 Sep 8;21(9):e1013427. doi: 10.1371/journal.pcbi.1013427 (PMC12435692; doi:10.1371/journal.pcbi.1013427)
Supplement: S1 Text [file pcbi.1013427.s001.pdf]

Supplementary Information

A Implementing SMoRe GloS

We describe how the five steps of SMoRe GloS were implemented for the two ABMs considered in this study. These are also summarized in Table A.

Step 1: Generate ABM output. For the *in vitro* cell proliferation ABM, we performed global sensitivity analysis using $d = 7$ parameters, each sampled 3 times on a regular grid. This yielded a total of 3^7 points in the ABM parameter space. For the 3D vascular tumor growth ABM, we selected $d = 4$ parameters for the global sensitivity analysis, again sampling each 3 times on a regular grid, resulting in 3^4 points in the ABM parameter space. In both cases, we averaged the results of $N = 6$ simulation runs for each sampled parameter vector.

Step 2: Formulate candidate surrogate models. For the *in vitro* cell proliferation ABM, our output metric of interest was total cell number at the end of the simulation. We therefore chose cell numbers in G1/S and G2/M phases of the cell cycle as the SM variables, and a system of two coupled ordinary differential equations (ODEs) describing their temporal evolution as the SM itself (see [1] for more details).

For the 3D vascular tumor growth ABM, our output metrics of interest were: (1) final tumor volume; (2) area under the tumor volume time-course; and (3) time to half-maximum tumor volume. We selected these metrics based on their ability to capture different aspects of the data simulated by the ABM. Specifically, the final tumor size is independent of the dynamic properties of the tumor volume time-course, such as its shape and curvature. In contrast, both the area under the curve and the time to half-maximum volume are influenced to different degrees by these properties. Since ABM output was being integrated over space in all three instances, we once again used ODEs to formulate the SM, taking total cell number as the SM variable. Three candidate SMs were formulated in this case, namely, exponential growth, logistic growth and von Bertalanffy growth (see [2] for more details). The SMs together with the corresponding ABMs are listed in subsection C of the main manuscript.

Step 3: Select a surrogate model. In both our examples, we used weighted Residual Sum of Squares (RSS) to quantify goodness-of-fit of the SMs under consideration. Further, we used the profile likelihood approach [3–5] to generate 95% confidence bounds on SM parameters, thereby quantifying uncertainty in SM parameter values. Identifiability indices were computed by graphing the likelihood curves obtained by profiling each fitted SM parameter. These cross the 95% confidence bound threshold never (a flat curve), once (an L-shaped curve), or twice (a U-shaped curve) in the neighborhood of its best-fit value. The respective identifiability index values are 0, 1 or 2.

For the *in vitro* cell proliferation ABM, we did not need to perform model selection since we started with a single SM. For the 3D vascular tumor growth ABM, the Akaike Information Criterion (AIC) in Eq A below was used to aid in model selection.

$$AIC \,=\, 2 \times (\#\text{ parameters}) \,+\, n \ln(\overline{\text{RSS}}), \tag{A}$$

where $\overline{RSS}$ is the average RSS taken over n data points. Models with higher ΔAIC scores are less likely to explain the data. To compare between models, we computed a

relative log-likelihood (RLL), defined as

$$RLL = \frac{1}{2}\left(AIC_{\text{model1}} - AIC_{\text{model2}}\right), \tag{B}$$

where a positive value of RLL indicates that model 2 is preferable to model 1.

Finally, we selected the logistic SM as the best candidate for the 3D vascular tumor growth ABM based on our criterion of maximizing goodness-of-fit while minimizing uncertainty in SM parameters, as indicated by a high frequency of 2's in their identifiability indices.

Step 4: Infer relationship between SM and ABM parameters. In both our examples, SM parameter hypersurfaces were generated as described in [2].

Step 5: Use relationship between surrogate model and ABM parameters to infer global sensitivity of ABM parameters. In both examples, we used two methods for global sensitivity analysis–MOAT and eFAST (see next section)–and employed the SM to approximate ABM output, including at points in the ABM parameter space where output was not generated in Step 1.

B 2D *In Vitro* Cell Proliferation ABM

We employ the easy-to-simulate ABM presented in [1, 6], which describes a 2-dimensional on lattice birth-death-migration model of cell proliferation. Cell division occurs as cells progress through four stages of the cell cycle in order: G1, S, G2, and M with transition rates, $\rho_{G1\to S}, \rho_{S\to G2}, \rho_{G2\to M}, \rho_{M\to G1}$, respectively. When a cell advances from M back to G1, it can proliferate into an unoccupied neighboring lattice site, provided the strength of contact inhibition on it is below a threshold T_{con}. Otherwise, the cell returns to G1 without undergoing mitosis. Cells move to neighboring lattice sites at a constant migration rate, s, provided a randomly selected neighboring lattice site is unoccupied. If not, cells remain stationary. The growth culture is assumed to have a carrying capacity K_A. For complete details on ABM formulation and simulation method, see [6].

C 3D Vascular Tumor Growth ABM

We employ the computationally complex model of vascular tumor growth in 3 dimensions presented in [7]. This on-lattice ABM consists of two modules that communicate with each other: a cancer cell module, governing tumor cell proliferation, migration and death; and a vascular module, governing vascular network growth and remodeling. The two modules are described in detail below.

The cancer cell module comprises cancer progenitor cells, which make up the bulk of the tumor, and cancer stem cells. The proliferation rate p_{div} of progenitor cells is greater than the proliferation rate s_{div} of cancer stem cells. Progenitor cells can divide a limited number of times, p_{lim}, before they become senescent. On the other hand, cancer stem cells have limitless replicative potential. Progenitor cells reproduce symmetrically to produce two daughter progenitor cells, whereas cancer stem cells can reproduce asymmetrically or symmetrically, producing a progenitor daughter cell and a stem cell, or two stem cells. Both types of cancer cells migrate or proliferate only if there is space in an adjacent lattice site (Moore's neighborhood). Both cell types are assumed to have a common migration rate, mig. A second factor governing the ability of a cancer cell to migrate or divide is its oxygen status, which could be normoxic (maximum migration and proliferation rates) or hypoxic (minimum migration and proliferation rates). This oxygen status is determined by the cell's distance from a mature, blood-borne vessel.

The second module comprises endothelial cells and simulates angiogenesis: the formation of new blood vessels within the tumor. The tumor initially starts with a mature vasculature along its boundaries. As the tumor grows past the diffusion threshold of oxygen, the cancer cells become hypoxic. This triggers an 'angiogenic-switch' and cancer cells begin secreting Vascular Endothelial Growth Factor (VEGF), initiating angiogenesis. In response to this chemical stimulus, mature vessels near a hypoxic cancer cell can sprout, forming a new (non-mature) vessel. This sprout proliferates, extends, and migrates up the gradient of VEGF towards the nearest hypoxic cells until it anatamoses (fuses with) another sprout or with a nearby mature vessel. Once anastamosis occurs, the sprouts involved become blood-borne (mature) and nearby cancer cells become normoxic. We refer the reader to [2] for complete details on this ABM and how to simulate this ABM.

D Computational efficiency of SMoRe GloS

Implementing the MOAT method directly with d parameters using a Latin Hypercube Sampling (LHS) of k points and n_r replicates at each point requires $(d+1) \times k \times n_r$ ABM simulations. The $d+1$ factor accounts for perturbing each LHS sample vector across all d parameter components. Typically, k values are recommended to range between 10 and 50 [8]. For the 3D vascular tumor growth ABM, we varied $d = 4$ parameters using $k = 15$ LHS points, with $n_r = 6$ replicates, requiring 450 ABM simulations. Each simulation lasted, on average, 10 minutes, resulting in a total wall time of approximately 75 hours when run serially. In contrast, with SMoRe GloS, we started with the same $(d+1) \times k = 75$ ABM parameter points, but we drew 100 samples from the corresponding surrogate model (SM) parameter subspaces for each. This produced a total of 7,500 SM simulations. Solving the SM has a negligible computational cost and SMoRe GloS completed this task in under one minute.

For the more computationally intensive eFAST method, we applied eFAST to $d = 4$ parameters, with $N_r = 2$ replicates per parameter (corresponding to random phase shifts), and $N_s = 65$ samples per curve. The value $N_s = 65$ is the minimum recommended [9]. As with the MOAT method, we ran $n_r = 6$ replicates at each point to estimate the average ABM behavior. This led to a total of $d \times N_r \times N_s \times n_r = 3,120$ ABM simulations, which, if run serially, would require nearly 22 days of wall time. In contrast, SMoRe GloS once again demonstrated its computational superiority by completing the eFAST analysis in under 5 minutes (Figure 5A, orange line in the main text).

To set up SMoRe GloS for the vascular tumor growth ABM, we sampled $g = 3$ points in each of the $d = 4$ dimensions of parameter space, with $n_r = 6$ replicates at each point, resulting in a total of $g^d \times n_r = 486$ ABM simulations. While this number is comparable to the simulations required for directly computing MOAT sensitivities, it is significantly lower than what would be required for directly implementing eFAST.

E Accuracy of SMoRe GloS

The accuracy of SMoRe GloS relies on the quality of the surrogate model parameter hypersurfaces. To ensure that these hypersurfaces capture important nonlinearities in the ABM parameter-response space, the initial sampling should be sufficiently fine-grained. In regions of high curvature, adaptive refinement of sampling density may further improve accuracy. To assess the reliability of the surrogate, we recommend cross-validation: ABM parameter vectors not used in constructing the hypersurfaces can be simulated and compared against the surrogate model predictions. In particular,

predicted profile likelihood intervals for surrogate model (SM) parameters can be compared to those derived from these withheld data points, providing a direct measure of hypersurface accuracy.

References

1. Bergman DR, Norton KA, Jain HV, Jackson T. Connecting Agent-Based Models with High-Dimensional Parameter Spaces to Multidimensional Data Using SMoRe ParS: A Surrogate Modeling Approach. Bulletin of Mathematical Biology. 2024;86(1):1–28.

2. Jain HV, Norton KA, Prado BB, Jackson TL. SMoRe ParS: A novel methodology for bridging modeling modalities and experimental data applied to 3D vascular tumor growth. Frontiers in Molecular Biosciences. 2022;9:1056461.

3. Venzon D, Moolgavkar S. A method for computing profile-likelihood-based confidence intervals. Journal of the Royal Statistical Society: Series C (Applied Statistics). 1988;37(1):87–94.

4. Eisenberg MC, Hayashi MA. Determining identifiable parameter combinations using subset profiling. Mathematical Biosciences. 2014;256:116–126.

5. Eisenberg MC, Jain HV. A confidence building exercise in data and identifiability: Modeling cancer chemotherapy as a case study. Journal of Theoretical Biology. 2017;431:63–78.

6. Bergman D, Jackson TL. Phenotype switching in a global method for agent-based models of biological tissue. Plos one. 2023;18(2):e0281672.

7. Norton KA, Jin K, Popel AS. Modeling triple-negative breast cancer heterogeneity: Effects of stromal macrophages, fibroblasts and tumor vasculature. Journal of Theoretical Biology. 2018;452:56–68.

8. Campolongo F, Cariboni J, Saltelli A. An effective screening design for sensitivity analysis of large models. Environmental modelling & software. 2007;22(10):1509–1518.

9. Marino S, Hogue IB, Ray CJ, Kirschner DE. A methodology for performing global uncertainty and sensitivity analysis in systems biology. Journal of Theoretical Biology. 2008;254(1):178–196.

Step	Description	2D	3D
1	Random sampling of ABM parameter space	grid	grid
2	Formulate candidate surrogate models	bespoke SM	exp, log, vB
3	Select best surrogate model	N/A	AIC and Identifiability Index
4	Infer SM-ABM relationship	SMoRe ParS	SMoRe ParS
5	Choose global sensitivity method(s)	MOAT and eFAST	MOAT and eFAST

Table A. Choices made in carrying out the steps of the SMoRe GloS workflow for the 2D *in vitro* cell proliferation ABM and the 3D vascular tumor growth ABM.

Name	Meaning	Range	Units
K_A	Carrying capacity	$[500, 1500]$	cells
T_{con}	Contact inhibition	$\{4, 5, 6\}$	cells
s	Cell migration rate	$[0, 20]$	$\mu\text{m}\,\text{d}^{-1}$
$\rho_{\text{G1}\to\text{S}}$	G1 $\to$ S transition rate	$1/11 \pm 10\%$	h^{-1}
$\rho_{\text{S}\to\text{G2}}$	S $\to$ G2 transition rate	$1/8 \pm 10\%$	h^{-1}
$\rho_{\text{G2}\to\text{M}}$	G2 $\to$ M transition rate	$1/4 \pm 10\%$	h^{-1}
$\rho_{\text{M}\to\text{G1}}$	M $\to$ G1 transition rate	$1 \pm 10\%$	h^{-1}

Table B. Parameter ranges sampled for Cell Proliferation ABM. Note, in the GitHub repository, the time unit used is days.

Name	Meaning	Range	Units
p_{div}	Progenitor proliferation rate	$[0.05, 0.245]$	probability in 6 h
s_{div}	Stem cell proliferation rate	$[0.01, 0.1]$	probability in 6 h
r_{mig}	Tip cell migration rate	$[1, 3]$	unitless
p_{lim}	Progenitor division limit	$8 - 15$	# divisions

Table C. Parameter ranges sampled for Vascular Tumor Growth ABM. Note, in the GitHub repository, the time unit used is days.

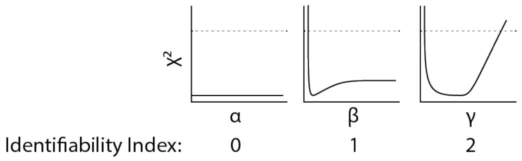

Fig A. Typical profiles of parameters with identifiability index 0 (α), 1 (β), and 2 (γ). The dashed lines indicates the 95% confidence bounds following the χ^2-distribution.

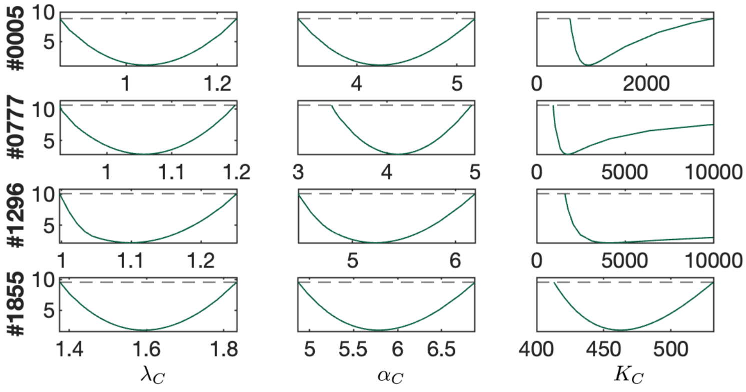

Fig B. Profile likelihoods of surrogate model (SM) parameters at four randomly selected ABM parameter vectors for the ABM representing a cell proliferation assay.

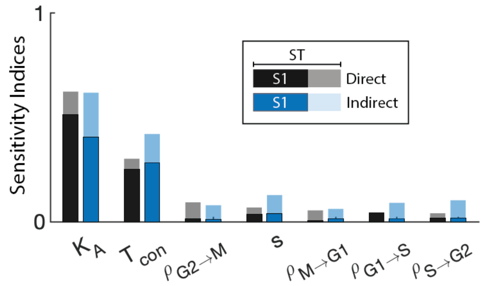

Fig C. SMoRe GloS recapitulates eFAST global sensitivity of final tumor volume using the surrogate model (SM). Compare with Figure 2. Solid color, black-bordered bars represent first-order sensitivity indices. Transparent bars represent total-order indices. Note: these are not stacked bar plots; the total-order index is given by the height of the transparent bar, not the difference with the height of the first-order index bar.

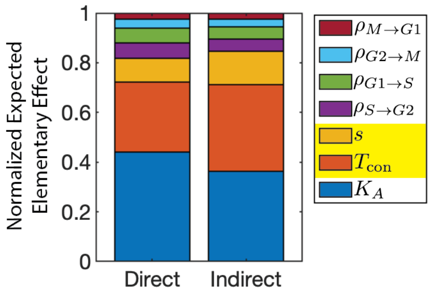

Fig D. Normalized MOAT sensitivity values for each ABM parameter in the cell proliferation assay model using the direct (left) and indirect (right) methods. Spatial parameters not not explicitly captured by the SM are highlighted in yellow.

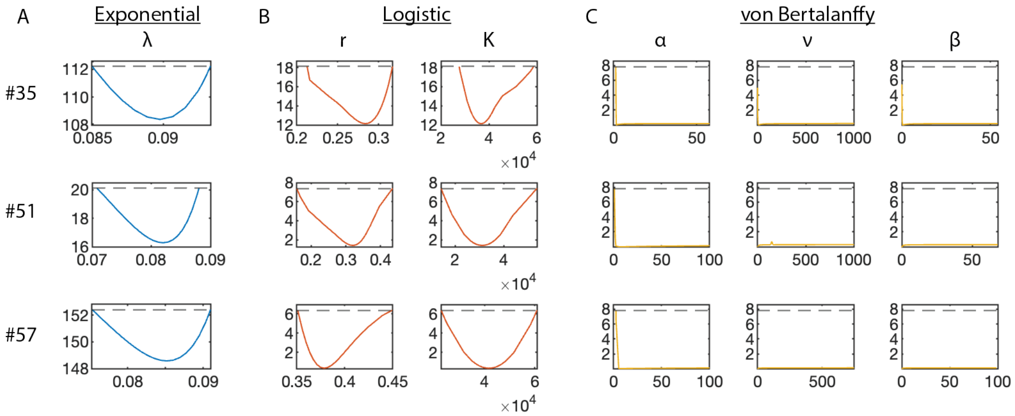

Fig E. Comparison of the identifiability properties of the three surrogate models (SMs) for approximating the 3D vascular tumor growth ABM. Profile likelihoods for three representative ABM parameter vectors (rows) for each SM parameter (columns).

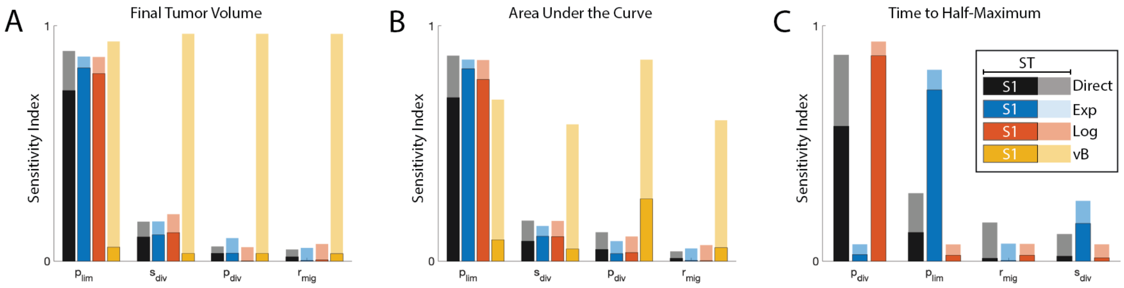

Fig F. SMoRe GloS recapitulates eFAST global sensitivity of multiple output ABM metrics using the logistic SM. Compare with Figure 4. Solid color, black-bordered bars represent first-order sensitivity indices. Transparent bars represent total-order indices. Note: these are not stacked bar plots; the total-order index is given by the height of the transparent bar, not the difference with the height of the first-order index bar.

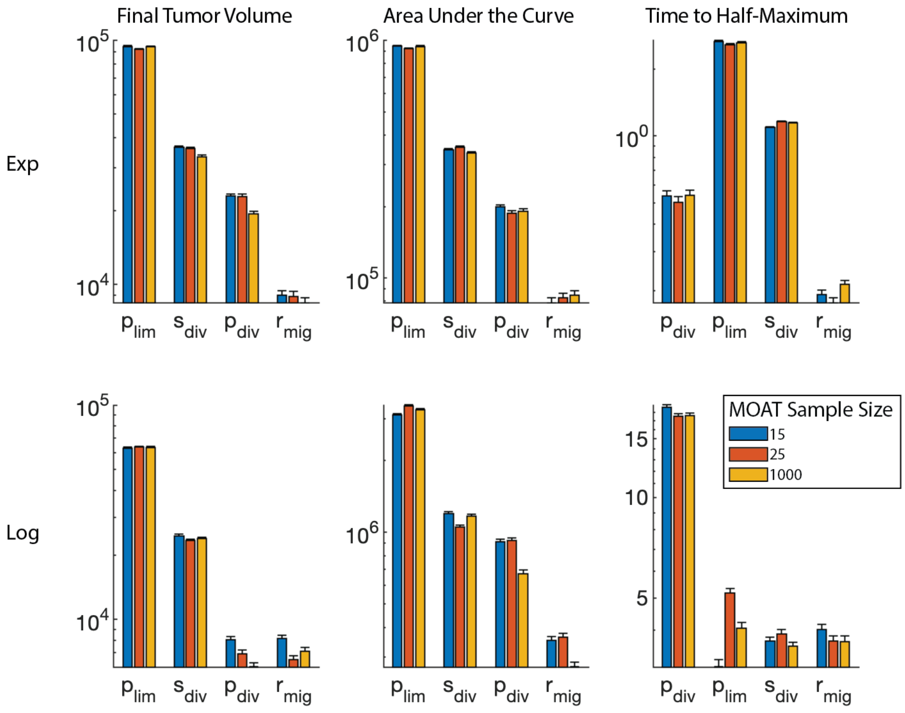

Fig G. SMoRe GloS MOAT sensitivity analyses rapidly converge. For each output ABM metric (columns) and with each surrogate model (rows), compute the MOAT for all four ABM model parameters using SMoRe GloS using 15 (blue bars), 25 (red bars), or 1000 (yellow bars).

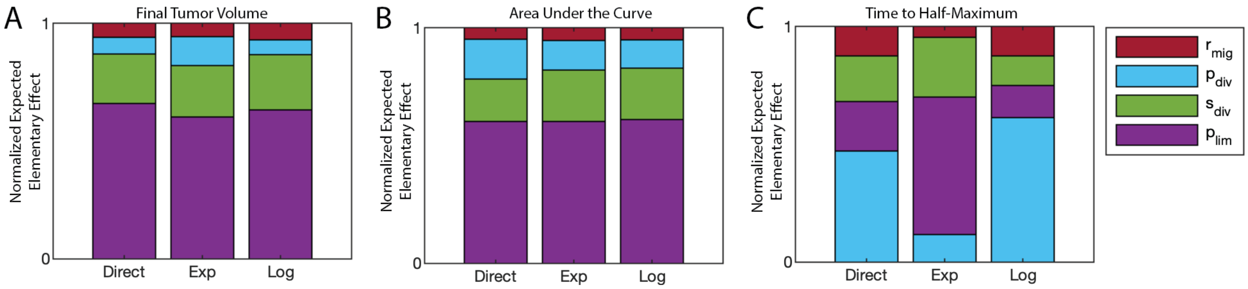

Fig H. Compare with Figure 4. Normalized MOAT sensitivities.
